# Physical activity and sedentary behavior across three time-points and associations with social skills in early childhood

**DOI:** 10.1186/s12889-018-6381-x

**Published:** 2019-01-07

**Authors:** Valerie Carson, Eun-Young Lee, Kylie D. Hesketh, Stephen Hunter, Nicholas Kuzik, Madison Predy, Ryan E. Rhodes, Christina M. Rinaldi, John C. Spence, Trina Hinkley

**Affiliations:** 1 Faculty of Kinesiology, Sport, and Recreation, University of Alberta, 8840-114 Street, Van Vliet Complex, Edmonton, AB T6G 2H9 Canada; 2 Institute for Physical Activity and Nutrition, School of Exercise and Nutrition Sciences, Faculty of Health, Deakin University, Geelong, VIC 3220 Australia; 3 School of Exercise Science, Physical and Health Education, University of Victoria, Victoria, BC V8W 2Y2 Canada; 4 Department of Educational Psychology, Faculty of Education, University of Alberta, Edmonton, AB T6G 2G5 Canada

**Keywords:** Toddlers, Physical activity, Sedentary behavior, Social skills, Accelerometer

## Abstract

**Background:**

The growth and development that occurs in early childhood has long-term implications, therefore understanding the relevant determinants is needed to inform early prevention and intervention. The objectives of the study were to examine: 1) the longitudinal associations of physical activity and sedentary behavior with social skills and 2) how physical activity and sedentary behavior track over three time-points.

**Methods:**

Participants were from the Parents’ Role in Establishing healthy Physical activity and Sedentary behavior habits (PREPS) project. A total of 251 eligible toddlers and their parents participated at baseline in 2014/15 (time 1; 1.6 ± 0.2 years) and a sub-sample participated at 1-year (time 2; *n* = 79; 2.7 ± 0.3 years) and 2-year (time 3; *n* = 77; 3.7 ± 0.4 years) follow-ups. Sedentary time (≤25 counts/15 s), light-intensity physical activity (LPA; 26–419 counts/15 s), and moderate- to vigorous-intensity physical activity (MVPA; ≥420/15 s) were objectively measured with wGT3X-BT ActiGraph accelerometers, and standardized for wear time. Parents reported their children’s screen time (television/video, video/computer games) at all three time-points. Parents also reported on children’s social skills using the Adaptive Social Behavior Inventory (ASBI) at time-points 2 and 3, and comply (e.g., cooperates; 10 items), express (e.g., joins play; 13 items), and disrupt (e.g., teases; 7 items) subscales were created by summing items. Generalized estimating equations (GEE) were conducted to address objective one. Tracking coefficients (low: β1 < 0.30; moderate: β1 = 0.30–0.59; moderate-high: β1 = 0.60-0.90; high: β1 > 0.9) were conducted using GEE to address objective two.

**Results:**

Across the study, screen time was negatively associated with express (b = − 0.068, 95%CI: -0.114, − 0.023) and comply (b = − 0.056; 95%CI: -0.094, − 0.018) scores and positively associated with disrupt scores (b = 0.004; 95% CI: 0.001, 0.006). Findings were similar for television/videos but less consistent for video/computer games. No associations were observed for physical activity. Screen time significantly tracked at moderate-high levels (β_1_ = 0.63; 95% CI: 0.45, 0.81), while all other behaviors tracked at moderate levels (β_1_ = 0.35–0.49; *p* < 0.01) over the three time-points.

**Conclusions:**

Screen time was unfavorably associated with social skills across early childhood. Furthermore, all behaviors tracked at moderate to moderate-high levels from toddler to preschool ages. Therefore, promoting healthy physical activity and sedentary behavior patterns early in life, especially for screen time, may be important.

## Background

Early childhood, defined as the first six years of life, is a critical life phase characterized by substantial growth and development in several domains, including the physical, social, emotional, and cognitive domains [1]. Young children are therefore malleable and their growth and development can be enhanced or impaired by early life experiences [1]. In terms of the social domain of development in early childhood, social competence has been identified as a key element [2]. Socially competent children are typically characterized as possessing the abilities to: “interact with others effectively” and “develop positive relationships” [2]. Within this complex construct, social skills have been identified as a central component as they are the specific abilities that underlie competence [3, 4]. Important long-term implications have been observed for social skills in early childhood. For instance, they have been linked with better education, employment, and mental health outcomes as well as lower criminal activity and substance use in adolescence and adulthood [5].

Given the important long-term implications of social skills in early childhood, it is important to understand the relevant determinants to inform early prevention and intervention [2]. A growing body of evidence suggests physical activity and sedentary behavior, in particular screen time, in early childhood are important predictors of multiple domains of development, including the social domain [6–8]. Play is considered critical for children’s development, and physically active play is considered a main category of play in early childhood [9]. Through play, young children learn and practice important social skills via interactions with parents, siblings, peers, and significant others, primarily in home and child care settings [10]. Conversely, screen time in early childhood is linked to decreased interactions with caregivers [11] and lower vocabulary [12], which are essential to developing social skills [2]. Furthermore, screen time may displace time spent in more developmentally enriching activities [13]. However, important research gaps and limitations have been highlighted in recent reviews examining physical activity, sedentary behavior, and the social domain of development in early childhood [6, 7]. For instance, to overcome limitations of previous studies, future longitudinal studies using objective measures of physical activity and sedentary behavior have been recommended [6]. Additionally, while important developmental processes occur in infanthood and toddlerhood (< 3 years) [2], most research, particularly investigating associations with physical activity and social skills, has focused on preschool-aged children (3–5 years) [7]. Therefore, studies with samples of children < 3 years are needed to fill this notable gap.

Another central reason for targeting physical activity and sedentary behavior in early childhood is the potential for behavioral patterns established early in life to remain stable overtime resulting in sustained impact on growth and development. For instance, moderate effect sizes have been observed for the tracking of both physical activity and sedentary behavior during early childhood and from early childhood to middle childhood [14]. There is some evidence that these health behaviors, in particular screen time, are associated with social skills in school-aged children and youth [15, 16]. However, a review on the tracking of physical activity and sedentary behavior found no studies on physical activity [14] and only two on sedentary behavior [17, 18] that included a sample of children < 3 years at baseline. Consequently, it is unclear if physical activity and sedentary behavior also track from toddler to preschool ages. Therefore, the objectives of this study were to examine: 1) the longitudinal associations of physical activity and sedentary behavior with social skills and 2) how physical activity and sedentary behavior track over three time-points in a sample of toddlers from Edmonton, Canada.

## Methods

### Participants

Participants were from the Parents’ Role in Establishing healthy Physical activity and Sedentary behaviour habits (PREPS) project. From October, 2014 to December, 2015, parents and their toddlers were recruited during routine 18-month immunization appointments at one of four public health centers in Edmonton, Canada. These health centers where chosen because they were large and busy centers that served diverse communities. To be eligible for the study, children needed to be ambulatory, and one parent needed to be comfortable speaking and reading in English. Of the 491 eligible families approached, 52% or 257 families agreed to participate in the baseline portion of the study. The reasons why families did not agree to participate are published elsewhere [19].

At baseline (time 1, 2014–2015) of the PREPS project, the majority of participating parents (*n* = 242 or 94%) indicated that they would be interested in being contacted for future research. In 2015, funding was secured to add 1-year (time 2, 2015–2016) and 2-year (time 3, 2016–2017) follow-ups to the PREPS project. Of the 242 eligible participants, 32 were not contacted at times 2 or 3 due to challenges associated with accelerometer return at time 1. Of the 210 remaining participants at time 2, 99 agreed to participate, 21 declined (17 agreed to stay on contact list for time 3 and four asked to be removed), 66 were not reachable (e.g., telephone or e-mail no longer working, no response after multiple attempts), and 24 were missed by error. Data were collected between October, 2015 and September, 2016. Of the 206 remaining participants at time 3, 92 agreed to participate, 30 declined, and 84 were not reachable. Data were collected between October, 2016 and December, 2017. Ethics approval was obtained from the University of Alberta Human Research Ethics Board and all participating parents provided written informed consent at all three time-points.

### Procedures

At time 1, eligible participating parents completed the PREPS questionnaire and were given an accelerometer and pre-paid courier return envelope during the 15-min wait period that is required after all immunizations. At times 2 and 3, after parents agreed to participate via phone or e-mail, study materials, including a follow-up PREPS questionnaire, accelerometer, and pre-paid courier return envelope were mailed to participants. At all three time-points, questionnaires were checked for missing data and parents were followed up when necessary. Additionally, mid-week reminders on the accelerometer procedures were provided to parents via e-mail. Finally, after the accelerometer was returned, a $25 gift certificate was mailed to families.

### Physical activity and sedentary behavior

At all three time-points, sedentary time and physical activity were measured with Actigraph wGT3X-BT accelerometers (ActiGraph Corp, Pensacola, FL, USA). Parents were informed that for the entire week their child should wear the accelerometer over their right hip, except during overnight sleep and water-based activities (e.g., swimming, bathing). Data were collected in 15-s epochs. For participants’ data to be included at each time-point, they were required to have ≥4 days with ≥1440 total 15 s intervals (equivalent to ≥6 h) of wear time. Previous studies have shown that these wear time parameters provide reliable estimates (Intra class correlation [ICC] = 0.70–0.80) of physical activity in toddlers and preschoolers [20, 21]. Non-wear time was defined as ≥80 consecutive 15-s intervals of zero counts (equivalent to ≥20 min of consecutive zeros counts) [22]. Daytime naps were assumed to be removed with non-wear time. For wear time data, sedentary time was defined as 0–24 counts per 15 s, light-intensity physical activity (LPA) as 25–420 counts per 15 s, and moderate- to vigorous-intensity physical activity (MVPA) as > 420 counts per 15 s. When compared to direct observation, these cut-points have shown fair to excellent validity in toddlers and preschoolers (Receiver operating characteristics – Area under the curve [ROC-AUC] = 0.72–0.90) [23, 24]. Minutes per day of sedentary time, LPA, and MVPA were calculated by dividing the number of 15-s intervals for sedentary time, LPA, and MVPA by 4 and then dividing by the total number of valid days. To adjust for wear time, standardized sedentary time, LPA, and MVPA variables were calculated at each time point [25].

At all three time-points, parents reported their toddler’s average screen time through four items in the PREPS questionnaire. Specifically, parents reported the average hours and minutes per weekday and weekend day that their toddler: 1) watches television, videos, or DVDs on a television, computer, or portable device; 2) plays video/computer games on devices such as a learning laptop, leapfrog leapster, computer, laptop, tablet, cell phone, the internet, Playstation, or XBOX. Weighted averages ([weekday*5 + weekend*2]/7) were calculated for television/videos and video/computer games variables, and minutes per day of toddlers’ screen time was calculated by summing the weighted averages. These screen time items were modified from a national survey in Canada [26], and had good 1-week test re-test reliability (Intra-class correlation [ICC] = 0.82) in a sub-sample of PREPS participants [27].

### Social skills

At times 2 and 3, parents reported their toddler’s social skills using the Adaptive Social Behavior Inventory (ASBI) [28, 29] as part of the PREPS questionnaire. Parents did not report on the ASBI at time 1 because the tool is considered developmentally appropriate for children aged approximately 2.5 to 5 years [29]. The ASBI includes 30 items, each with three response options (“rarely/never,” “sometimes,” or “always”), which form three sub-scales: express (e.g., understands others’ feelings; will join a group of children playing; 13 items), comply (e.g., helpful to other children; is calm and easy going; 10 items), and disrupt (e.g., teases other children; is bossy; 7 items) [28, 29]. Items were summed to create each sub-scale score. In this sample, internal consistency reliability was α = 0.79, 0.81, 0.65 at time 2 and α = 0.78, 0.76, 0.53 at time 3 for express, comply, and disrupt sub-scales, respectively. The lower alpha for the disrupt score is consistent with the study that developed the ASBI [28]. Specifically, due to the low number of items in the disrupt score with factor loadings of ≥0.40, two items with factor loadings of ≥0.35 were included [28].

### Covariates

Based on previous research [6–8], child age at all three time-points, child sex at time 1, and parental education at time 1 were included as covariates in the analyses. Questionnaire completion date at times 2 and 3, child birthdate, child sex, and parental education were reported by parents as part of the PREPS questionnaire. Child age (years) was calculated by subtracting the exam date (time 1) or questionnaire completion dates (times 2 and 3) from the birthdate. Parent education was the highest grade or level of education of the parent completing the questionnaire. There were six response options ranging from “no schooling” to “post-graduate”.

### Statistical analyses

Statistical analyses were completed using SAS version 9.4 (SAS Institute Inc., Cary, NC). Descriptive statistics were calculated, and differences on time 1 demographic, physical activity, and sedentary behavior variables between participants included and excluded at time 2 and 3 were examined using t-tests or Wilcoxon Rank Sum tests and chi-squared statistics. To examine the longitudinal associations of each physical activity and sedentary behavior variable with social skills, generalized estimating equations (GEE) were conducted that adjusted for child age, child sex (time 1), and parental education (time 1). GEE uses all available data, to produce a single regression coefficient, which represents pooled cross-sectional (between-subjects) and longitudinal (within-subject) associations [30]. To correct for the non-independent repeated measures data, GEE uses an a priori determined correlation structure [30]. An exchangeable correlation structure was used for all models addressing objective one [30]. Sedentary behavior and physical activity variables were expressed as 10 min/day within these models to make the interpretation of the regression coefficients more meaningful. The disrupt time 2 and 3 variables were log transformed to meet the assumption of normality. When observations were identified as potential influential cases by examining Cook’s distance values and data entry errors were ruled out, models were run with and without those observations to determine if findings differed.

To examine how physical activity and sedentary behavior track over three time-points, longitudinal tracking coefficients (β_1_) were calculated using GEE [30]. Z-scores were first calculated for sedentary behavior and physical activity variables so a standardized longitudinal tracking coefficient could be obtained. Then the time 1 sedentary behavior or physical activity variable was regressed on the corresponding longitudinal sedentary behavior or physical activity variables from time 2 to time 3 [30]. An unstructured correlation structure was used for all models addressing objective two [30]. Tracking coefficients were defined as low (β_1_ < 0.30), moderate (β_1_ = 0.30–0.59) moderate-high (β_1_ = 0.60–0.90), and high (β_1_ > 0.9) [31]. Statistical significance was defined as *p* < 0.05.

## Results

Of the 257 participants at time 1, all participants provided questionnaire data and 155 had complete accelerometer data. Of the 99 participants at time 2, 84 provided questionnaire data and 65 provided complete accelerometer data. Of the 92 participants at time 3, 83 provided questionnaire data and 63 provided complete accelerometer data. Two participants were excluded because they were > 36 months at baseline and four participants were excluded because they had a disability that limited their ability to be physically active. Furthermore, television/videos, video/computer game, and screen time values for three participants were set to missing as they had unrealistic values (e.g., 29 h/day).

Participant characteristics are shown in Table 1. No significant differences existed for child age, child sex, parental education, sedentary time, LPA, MVPA, screen time, television/videos, video/computer games at baseline between the included and excluded samples at time 2 and time 3.

**Table 1:** Participant Characteristics

Demographic variables	Time 1 (2014/2015) (*n* = 251)	Time 2 (2015/2016) (*n* = 79)	Time 3 (2016/2017) (*n* = 77)
Children’s characteristics			
Age (years)	1.6 (0.2)	2.7 (0.3)	3.7 (0.4)
Sex			
Male	50.6	–	–
Female	49.4	–	–
Parental characteristics			
Age (years)	33.4 (5.0)	–	–
Sex			
Male	13.9	14.1	7.8
Female	86.1	85.9	92.2
Highest level of education (*n* = 250)			
High school (grades 9–12)	14.4	–	–
Community/Technical college	27.2	–	–
University	38.0	–	–
Post-graduate	20.4	–	–

Values represent mean (standard deviation) for continuous values, and percentage for categorical values

A missing value means this question was not asked at the respective time point

The associations of sedentary time, LPA, MVPA, screen time, television/videos, and video/computer games with social skills over three time-points are displayed in Table 2. Higher screen time was significantly associated with lower express (b = − 0.068, 95% CI: -0.114, − 0.023) and comply (b = − 0.056; 95% CI: -0.094, − 0.018) scores and higher disrupt scores (b = 0.004; 95% CI: 0.001, 0.006). Similar findings were observed for television/videos and express (b = − 0.069; 95% CI: -0.125, − 0.013), comply (b = − 0.075; 95% CI: -0.134, − 0.016), and disrupt (b = 0.007; 95% CI: 0.004, 0.010) scores as well as video/computer games and comply scores (b = − 0.085; 95% CI: -0.159, − 0.011). However, associations between video/computer games and express were no longer significant when one observation was removed based on its Cook’s distance value. No significant associations were observed between accelerometer-derived sedentary time, LPA, or MVPA, and social skills.

**Table 2:** Associations of sedentary behavior and physical activity with social competence over three time-points

	Express		Comply		Disrupt^b^	
	b (95% CI)	*p*-value	b (95% CI)	*p*-value	b (95% CI)	*p*-value
Accelerometer-derived						
Sedentary time (10 min/day)^a^	− 0.083 (− 0.182, 0.017)	0.105	0.018 (− 0.080, 0.115)	0.722	0.004 (− 0.004, 0.011)	0.312
LPA (10 min/day)^a^	0.129 (− 0.023, 0.282)	0.097	0.001 (− 0.150, 0.151)	0.992	− 0.005 (− 0.014, 0.005)	0.339
MVPA (10 min/day)^a^	0.069 (− 0.201, 0.338)	0.619	0.013 (− 0.170, 0.196)	0.891	−0.002 (− 0.016, 0.017)	0.738
Parental-reported						
Screen time (10 min/day)	**−0.068 (− 0.114, − 0.023)**	**0.003**	**−0.056 (− 0.094, − 0.018)**	**0.002**	**0.004 (0.001, 0.006)**	**0.007**
Television/videos (10 min/day)	**− 0.069 (− 0.125, − 0.013)**	**0.016**	**−0.075 (− 0.134, − 0.016)**	**0.013**	**0.007 (0.004, 0.010)**	**< 0.001**
Video/computer games (10 min/day)	**− 0.117 (− 0.222, − 0.012)** ^**c**^	**0.030**	**−0.085 (− 0.159, − 0.011)**	**0.025**	0.001 (− 0.005, 0.006)^d^	0.827

*PREPS* Parents’ Role in Establishing healthy Physical activity and Sedentary behaviour habits. This project took place in Edmonton, Canada

*b (95% CI)* Unstandardized beta coefficients and 95% confidence intervals; *min/day* minutes per day

^a^Standardized for wear time

^b^Disrupt values at time 2 and time 3 were log-transformed

^c^Association was no longer significant (*p* = 0.09) when one influential observation was removed based on the Cook’s distance value

^d^Association approached significance (*p* = 0.09) when eight observations were removed based on the Cook’s distance value

***p*** **< 0.05**

Sedentary time, LPA, MVPA, screen time, television/videos, video/computer games over the three times-points are displayed in Table 3. Descriptively, the duration of all behaviors increased over time. The largest increase between time 1 and time 3 was observed for screen time, where there was a 48 min/day median increase. Screen time significantly tracked at moderate-high levels (β1 = 0.63; 95% CI: 0.45, 0.81), whereas, the other behaviors all significantly tracked at moderate levels (β1 = 0.35–0.49) over the three time-points.

**Table 3:** Sedentary behavior and physical activity over three time-points

	Time 1^b^	Time 2^b^	Time 3^b^	b (95% CI)	*p*-value
Accelerometer-derived					
Sedentary time (min/day)^a^	318.4 (41.6)	334.2 (50.0)	352.1 (47.2)	**0.44 (0.26, 0.62)**	**< 0.001**
LPA (min/day)^a^	237.8 (29.3)	259.1 (35.6)	273.2 (30.2)	**0.35 (0.17, 0.54)**	**< 0.001**
MVPA (min/day)^a^	59.0 (19.7)	73.2 (24.6)	89.8 (29.1)	**0.49 (0.33, 0.66)**	**< 0.001**
Parental-reported					
Screen time (min/day)	71.8 (18.9–154.3)	94.3 (50.5–197.1)	120.0 (53.6–188.6)	**0.63 (0.45, 0.81)**	**< 0.001**
Television/videos (min/day)	60.0 (15.4–122.1)	81.4 (41.8–173.6)	87.9 (50.4–147.9)	**0.49 (0.33, 0.64)**	**< 0.001**
Video/computer games (min/day)	0.0 (0.0–8.6)	0.0 (0.0–25.7)	5.7 (0.0–38.6)	**0.45 (0.18, 0.72)**	**0.001**

^a^Standardized for wear time

^b^Values represent mean (standard deviation) for variables normally distributed and median (inter-quartile range) for variables not normally distributed

***p*** **< 0.05**

## Discussion

This study addresses important gaps and limitations regarding physical activity and sedentary behavior in early childhood and associations with social skills using a longitudinal sample of children who were toddlers at baseline. The significant longitudinal associations observed between screen time and social skills suggests that screen time may be detrimental to social skills in early childhood. These observed associations appeared to be largely driven by television/video viewing, given it made up the majority of the total screen time at all three time-points. However, it is important to note that observed associations were small. On the other hand, objectively-measured physical activity and sedentary time were not significantly associated with social skills. Nonetheless, all behaviors, whether objectively-measured or parental-reported, were found to significantly track at moderate to moderate-high levels between toddler and preschool ages across the three time-points.

Within recent systematic reviews, no included study examined the relationship between accelerometer-derived sedentary time and social skills [6, 8], highlighting an important gap addressed by the present study. Among the nine longitudinal studies included in the most recent review, mainly unfavourable or null associations were observed between television viewing and social development, primarily over two time-points [8]. This study strengthens the evidence base by also reporting unfavourable associations for multiple types of screen time within a stronger study design that included three time-points. It is also important to note that within the review a wide variety of social development measures were used and not all of these measures were directly assessing social skills [8]. The ASBI measure, in comparison, was specifically developed to assess social competence, in particular social skills, in young children [29]. A cross-sectional study published after the most recent review, using the ASBI, also found that higher television/DVD/video viewing was associated with higher comply scores among 575 Australian children aged 2–5 years [32]. However, in contrast to the present study, associations were not observed with express and disrupt scores or when computer/e-game/hand-held game was the exposure [32]. Future longitudinal studies with multiple time points and experimental studies are needed to confirm and build on our findings.

Based on the findings of this study and previous evidence, what young children are doing while sedentary may be more important for social skills than the total time spent sedentary. Screen time in early childhood is a controversial topic. There is a multi-million dollar screen time industry that is marketed to parents of young children [13]. Consequently, studies have found it is common for parents of young children to think screen time is an appropriate learning tool or beneficial for cognitive development [33, 34]. However, several countries have recently developed evidence-based guidelines that do not support this point of view, and recommend no screen time for children under 2 years of age and one hour or less per day for children 2–4 years or 2–5 years, depending on the country [35–37]. It is important to note that the majority of evidence that informed these guidelines is based on television viewing using a traditional television [8]. As technology has evolved, newer electronic screen-based devices (e.g., smart phones, tablets) can also be used for passive television/video viewing and for interactive games. Though evidence is quite limited, interactive games may have different implications on cognitive development than that of passive television viewing [38]. However, the findings of the present study, which incorporated newer devices, suggest regardless of whether young children are using traditional or new devices, they are still primarily engaging in screen time through television/video watching, which is more likely to be passive compared to playing interactive games. This finding is supported by another recent study that reported on the activities young children liked when engaging with touch screens [39]. More research should examine if children’s interactions with caregivers and peers differ between passive and interactive screen time engagement to better understand the role that different types of screen time may have on the development of social skills.

In contrast to screen time, accelerometer-derived LPA and MVPA were not significantly associated with social skills in the present study. In a recent review on physical activity and health indicators in children aged 0–4 years [7], limited experimental evidence of moderate quality was found that suggests physical activity among preschoolers, in particular dance, is favourably associated with overall social competence [40]. However, no included longitudinal studies were reported in the review that included a measure of social competence or specifically social skills [7]. A cross-sectional study published after the most recent review found that outdoor play had favourable associations with the express and comply scores of the ASBI [32]. Therefore, similar to sedentary behavior, it could be that the specific types of physical activity and the context surrounding the physical activity engagement is more important for the development of social skills than total duration and intensity of physical activity. Since no previous studies to our knowledge have examined the associations of accelerometer-derived physical activity with social skills in this age group, future research is needed to confirm our findings around total duration and intensity.

Though physical activity was not associated with social skills, LPA and MVPA did track at moderate levels across the three time-points, with a stronger tracking coefficient being observed for MVPA compared to LPA. To our knowledge this is the first study to examine tracking of objectively-measured LPA and MVPA from toddler to preschool ages, though similar findings have been observed for counts per minute in a similar age group [41]. Findings also align with studies in older age groups that observed tracking of physical activity from early childhood to middle childhood [14]. Given that physical activity, MVPA in particular, is associated with a wide variety of health indicators throughout childhood [16], current evidence supports the promotion of regular physical activity, especially MVPA, in toddlerhood. Similarly, screen time was also found to significantly track over the three time-points, and it had the strongest tracking coefficient among all the behaviors examined. Previous studies have found that television viewing tracked at moderate levels (*r* = 0.54) from 18 to 30 months [18] and before age 3 years and between 3 and 5 years [17]. Therefore, overall findings across studies also highlight the importance of targeting screen time engagement early in life.

Main strengths of this study include the longitudinal design with three time-points, the toddler-aged sample at baseline, the objective measures of physical activity and sedentary time, and the inclusion of multiple types of screen time. A main limitation of the study is the loss of participants to follow-up. Some loss to follow-up is likely explained by PREPS originally being a cross-sectional study. Therefore, parents who agreed to participate at baseline did not consent to participate in future time-points. Nevertheless, no significant differences were observed for baseline demographic and behavior variables between included and excluded samples. Another limitation is the parental-reported screen time and social skills measures, which may have been impacted by biases, such as recall and social desirability. However, good reliability has been established for the screen time measure [27]. Additionally, the comply and express constructs had good reliability in the present sample, though consistent with previous research [28] the reliability for the disrupt construct was lower. Finally, given this was an observational study, residual confounding cannot be ruled out.

## Conclusions

Findings from this study suggest that screen time may be detrimental for social skills in early childhood. However, observed associations were small. Additionally, physical activity and sedentary behavior were found to track at moderate to moderate-high levels from toddler to preschool ages. Therefore, promoting healthy physical activity and sedentary behavior patterns early in life, in particular around screen-based sedentary behavior, may be important. Future longitudinal and experimental studies are needed to confirm our findings.

## Abbreviations

ASBI: Adaptive Social Behavior Inventory
GEE: generalized estimating equations
ICC: intra class correlation
LPA: light-intensity physical activity
MVPA: moderate- to vigorous-intensity physical activity
PREPS: Parents’ Role in Establishing healthy Physical activity and Sedentary behavior habits
ROC-AUC: receiver operating characteristics – area under the curve
